# Purifying selection shapes the coincident SNP distribution of primate coding sequences

**DOI:** 10.1038/srep27272

**Published:** 2016-06-03

**Authors:** Chia-Ying Chen, Li-Yuan Hung, Chan-Shuo Wu, Trees-Juen Chuang

**Affiliations:** 1 Genomics Research Center, Academia Sinica, Taipei 11529, Taiwan

## Abstract

Genome-wide analysis has observed an excess of coincident single nucleotide polymorphisms (coSNPs) at human-chimpanzee orthologous positions, and suggested that this is due to cryptic variation in the mutation rate. While this phenomenon primarily corresponds with non-coding coSNPs, the situation in coding sequences remains unclear. Here we calculate the observed-to-expected ratio of coSNPs (coSNP_*O/E*_) to estimate the prevalence of human-chimpanzee coSNPs, and show that the excess of coSNPs is also present in coding regions. Intriguingly, coSNP_*O/E*_ is much higher at zero-fold than at nonzero-fold degenerate sites; such a difference is due to an elevation of coSNP_*O/E*_ at zero-fold degenerate sites, rather than a reduction at nonzero-fold degenerate ones. These trends are independent of chimpanzee subpopulation, population size, or sequencing techniques; and hold in broad generality across primates. We find that this discrepancy cannot fully explained by sequence contexts, shared ancestral polymorphisms, SNP density, and recombination rate, and that coSNP_*O/E*_ in coding sequences is significantly influenced by purifying selection. We also show that selection and mutation rate affect coSNP_*O/E*_ independently, and coSNPs tend to be less damaging and more correlated with human diseases than non-coSNPs. These suggest that coSNPs may represent a “signature” during primate protein evolution.

Nucleotide mutation is thought to be the ultimate driving force of evolution. The processes that determine mutation rate may be highly complex and incompletely understood. In exonic regions (particularly in coding regions), mutations may have deleterious effects, and thus be prone to be eliminated in natural selection^1^. Therefore, investigation of single nucleotide polymorphisms (SNPs) in coding regions may offer unique opportunities to understand their cause and effect on diseases and evolution, and to decipher the cryptic mechanism underneath.

Previous studies have showed that there is an excess of coincident SNPs (coSNPs) between human and chimpanzee, which are human-chimpanzee orthologous sites observed to have a SNP in both species^2^,^3^. This observation cannot be fully explained by the CpG effect, GC content, simple contextual effects (such as effects of neighboring nucleotides), shared ancestral polymorphisms, natural selection, or technical artifacts, leaving a cryptic nature of mutation rate as the most likely explanation for this bias^2^,^3^,^4^,^5^. Nevertheless, the trends observed in genome-wide analyses are biased toward non-coding SNPs, as the vast majority of observed SNPs are located within non-coding regions. For example, 99.4% (1,507,949/1,517,605) of chimpanzee SNPs from dbSNP (Build 136) are non-coding SNPs. coSNPs in coding regions (designated as “coding coSNPs”) is relatively uninvestigated.

Since coding regions (particularly zero-fold degenerate nucleotides) are generally under stronger selection pressure than non-coding regions^6^, genetic variation in coding regions is rare, as a result of constraint by natural selection in a population. Several questions remain to be answered: (i) is the excess of coSNPs also present in coding regions? (ii) is the distribution of coSNPs dependent on the level of degeneracy (particularly zero-fold vs. nonzero-fold degenerate sites)? (iii) do the observed trends remain true in broad generality across primates? (iv) what is the most likely explanation for the distribution of coSNPs within coding regions? and (v) do coding coSNPs correlate with single-nucleotide substitutions and the fitness effects of amino acid substitutions?

To address these questions, we first sequenced the exomes of six unrelated western chimpanzees (*Pan troglodytes verus*), and identified 11,171 SNPs in coding regions. By comparing the known SNPs of human with these SNP datasets of chimpanzee, we identified human-chimpanzee coSNPs, and subsequently calculated the observed-to-expected ratio of coSNPs (coSNP_*O/E*_) to estimate the prevalence of coSNPs. Since population size is highly associated with the evolutionary dynamics of weakly-selected mutations^7^, we also controlled for this variable in our analysis. Our results showed that coding regions also contained an excess of coSNPs, and zero-fold degenerate sites had a greater enrichment of coSNPs than nonzero-fold degenerate sites. We showed that these observations held in broad generality across primates, and appeared independent of chimpanzee subpopulation, population size, and sequencing techniques. Next, we looked for possible explanations for the unexpected discrepancy of coSNP_*O/E*_ between zero-fold degenerate sites and nonzero-fold degenerate sites. After rejecting the possible explanations including sequence contexts, shared ancestral polymorphisms, density of single SNPs, and recombination rate, we showed that: (i) the strength of selective constraints was positively correlated with coSNP_*O/E*_ at zero-fold degenerate sites; (ii) the level of discrepancy of coSNP_*O/E*_ between zero-fold degenerate sites and nonzero-fold degenerate sites increased with increasing the strength of selective constraints; and (iii) selection and mutation rate affected coSNP_*O/E*_ independently. We thus concluded that purifying selection is important in shaping the distribution of coSNPs in coding sequences. Furthermore, we found that coSNPs were less deleterious, under more relaxed selection pressure, and more correlated with human diseases than non-coincident SNPs (designated as “non-coSNPs”; they are human-chimpanzee orthologous sites that were observed to be polymorphic in only one of the two compared species) at zero-fold degenerate sites. These observations indicate that selective constraints have been imposed on coding coSNPs, suggesting an important role of coSNPs during primate protein evolution.

## Results

### Coding regions also contain an excess of coSNPs

We sequenced the exomes of six unrelated western chimpanzees (designated as “CE6”) using SOLiD 4 System (Life Technologies, USA), and then used Novoalign (Novocraft Technologies) to align the color-space reads against the chimpanzee reference genome (PanTro 3). We found that 53.7%~64.8% of reads were uniquely mapped to the chimpanzee genome, and the average read coverage depth was greater than 45-fold (Supplemental Table S1). To ensure the accuracy in SNP calling, we only considered the human-chimpanzee orthologous consensus coding sequences (CCDSs)^8^ with sufficient read coverage (≥8× coverage; Supplemental Table S2) and outside of copy-number variations and repetitive regions (see Supplemental Fig. S1 and METHODS). Eventually, we identified 11,171 coding SNPs from the six chimpanzees (Table 1), 86% (9,615) of which were previously uncharacterized in the chimpanzee dbSNP (Build 136).

To reduce the potential issues of biological or technical biases, we also downloaded five chimpanzee SNP datasets from: the chimpanzee dbSNP dataset (Build 136; designated as “CdbSNP”), an exome sequencing dataset of 12 chimpanzees (designated as “CE12”)^9^, and three whole-genome sequencing datasets of 25 chimpanzees (designated as “CW25”)^10^, 10 chimpanzees (designated as “CW10”)^11^, and 5 chimpanzees (designated as “CW5”)^12^ (Table 1). Therefore, the chimpanzee SNP datasets analyzed in this study consisted of different chimpanzee subpopulations (western and central chimpanzees), different sequencing protocols (exome and whole-genome sequencing), and different sequencing platforms (SOLiD and Illumina sequencers) (Table 1).

**Figure 1:**
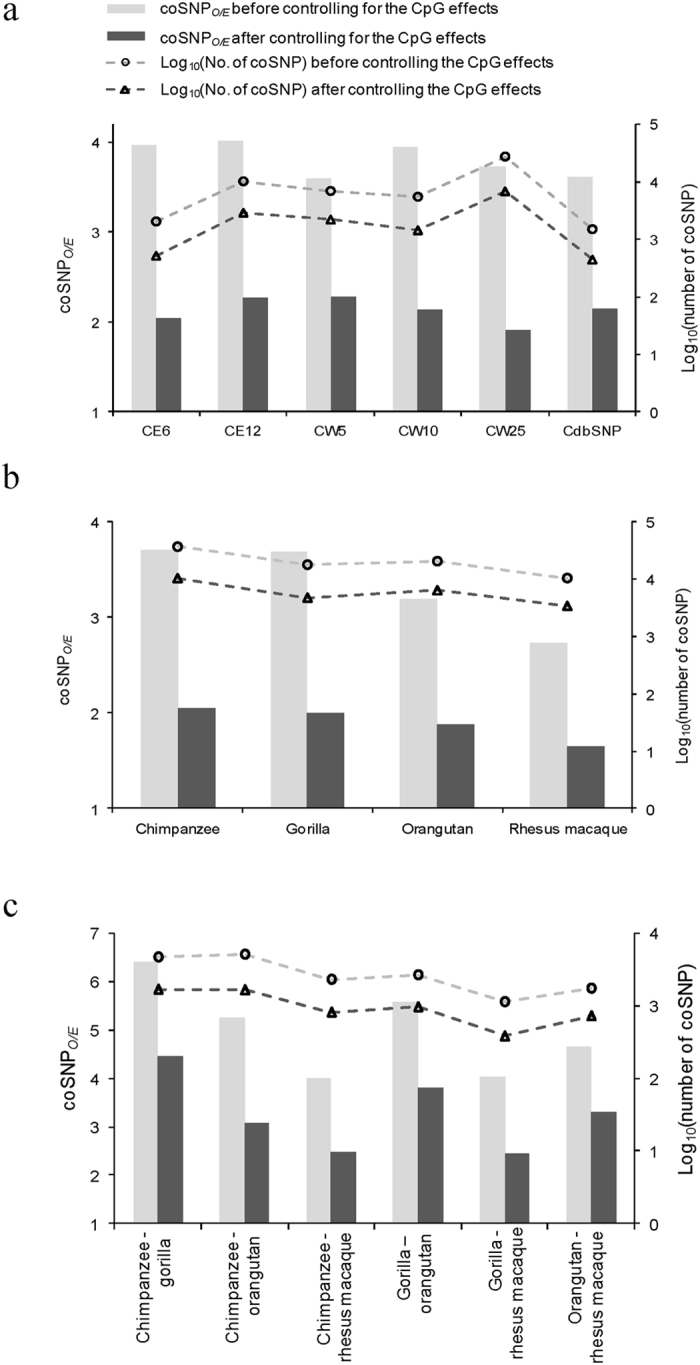
The coSNP_*O/E*_ and the corresponding logarithm values of the coding coSNPs (**a**) between human SNPs (dbSNP138) and each of six chimpanzee SNP datasets (CE6, CE12, CW5, CW10, CW25, and CdbSNP), (**b**) between human SNPs (dbSNP138) and SNPs of non-human primates (chimpanzee, gorilla, orangutan, and rhesus macaque), and (**c**) between SNPs of non-human primates before/after controlling for the CpG effect. The chimpanzee SNPs used in (**b**,**c**) are the combination of the six datasets listed in Table 1. The SNP datasets of gorilla, orangutan, and rhesus macaque are described in Supplemental Table S3. All the coSNP_*O/E*_ values are significantly greater than 1 (all *P* values < 10^−15^ by the *Chi*-square independence test).

Comparisons of the human SNPs (dbSNP Build 138) with each of the six chimpanzee SNP datasets allowed the extraction of human-chimpanzee coSNPs in coding regions (Table 1). We subsequently estimated the prevalence of coSNPs based on the observed-to-expected ratio of coSNPs (coSNP_*O/E*_; see METHODS). If SNPs were randomly distributed in both genomes of human and chimpanzee, the value of coSNP_*O/E*_ should be close to 1. We showed that all the coSNP_*O/E*_ values are significantly greater than 1 (all *P* values < 10^−15^ by the *Chi*-square independence test; Fig. 1a), indicating that the previous observation of coSNP enrichment in a whole-genome scale^2^ also holds true in coding regions alone. We emphasized that such a trend was independent of chimpanzee subpopulation, sequencing protocol, and sequencing platform, indicating that the observed trend was not a consequence of bias in the selection of SNP datasets.

Since coSNPs at CpG dinucleotides were observed to be of higher frequency of occurrence than non-coSNPs^13^, we excluded coSNPs located at CpG dinucleotides and showed that the enrichment of coding coSNPs still held (Fig. 1a). This indicated that the CpG effect could not be solely responsible for this enrichment. We proceeded to retrieved SNP data from other primates, namely orangutan, gorilla, and rhesus macaque (Supplemental Table S3), and examined the coSNP_*O/E*_ between human SNPs and SNPs of non-human primates (Fig. 1b), and the coSNP_*O/E*_ between SNPs of non-human primates (Fig. 1c). Such an excess of coSNPs in coding regions was present in all examined cases, and cannot be solely explained by the CpG effect.

### Degeneracy significantly affects the distribution of coding coSNPs

**Figure 2:**
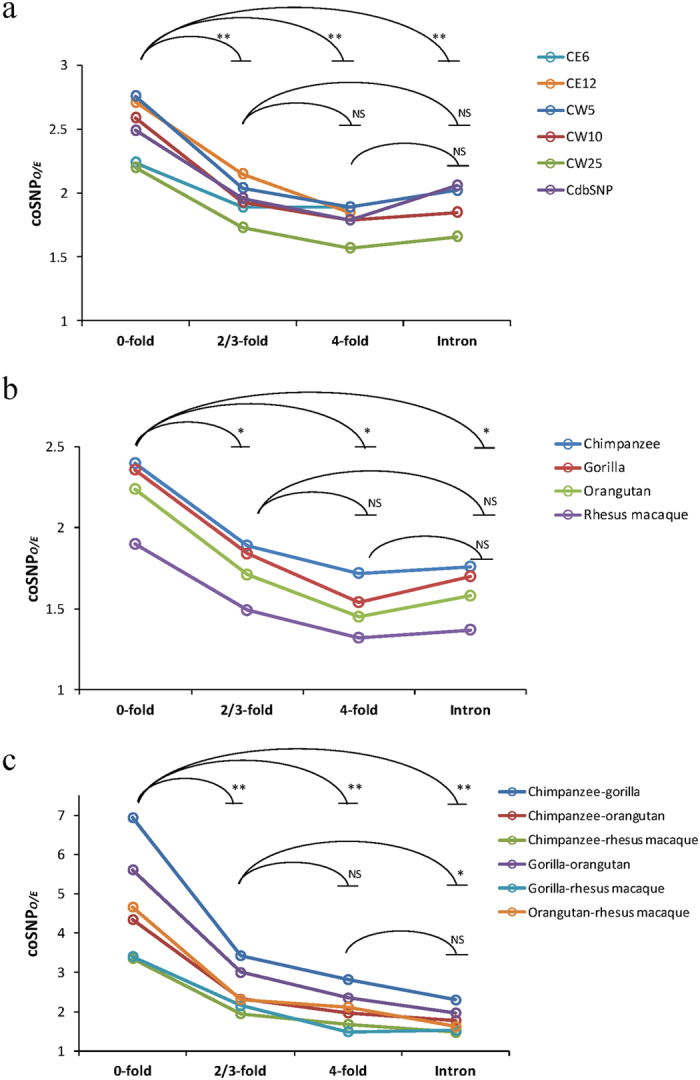
Comparisons of coSNP_*O/E*_ of different types of *i*-fold degenerate sites and intronic sequences based on the coSNPs (**a**) between human SNPs (dbSNP138) and each of the six chimpanzee SNP datasets (CE6, CE12, CW5, CW10, CW25, and CdbSNP), (**b**) between human SNPs (dbSNP138) and SNPs of non-human primates (chimpanzee, gorilla, orangutan, and rhesus macaque), and (**c**) between SNPs of non-human primates. The SNPs of non-human primates used in (**b**,**c**) are the same as in Fig. 1. For CE6 and CE12, intronic coSNP_*O/E*_ are not available, as the two datasets only contain exonic SNPs. *P* values were determined by the two-tailed Wilcoxon rank-sum test. Significance: *P < 0.05 and **P < 0.01. NS, not significant.

Since coding nucleotides with different levels of degeneracy are subject to different degrees of selective constraint^6^, we investigated whether the degeneracy of coding nucleotides is associated with the distribution of coSNPs. For the purpose, we separated the human-chimpanzee coSNPs into zero-fold (*i* = 0), two-/three-fold (*i* = 2 or 3), and four-fold (*i* = 4) degenerate sites (Table 2), and calculated the coSNP_*O/E*_ for each type of sites. We excluded the coSNPs at CpG dinucleotides in the following analysis, because they are essentially more mutagenic than other sites^3^. Figure 2a showed that the significant excess of human-chimpanzee coSNPs was present in all types of degenerate sites across different chimpanzee SNP datasets (all *P* values < 10^−15^). Interestingly, we noticed that coSNP_*O/E*_ was significantly higher at zero-fold than at nonzero-fold (i.e., two-/three-fold and four-fold) degenerate sites (both *P* values < 0.01 by the two-tailed Wilcoxon rank-sum test; Fig. 2a), suggesting that the degeneracy of nucleotides might be an indicator of selective constraints that could influence the distribution of coSNPs.

We then examined whether the coSNP_*O/E*_ was elevated at zero-fold degenerate sites, or reduced at nonzero-fold degenerate sites. We retrieved coSNPs located in human-chimpanzee orthologous introns by comparing human SNPs and four chimpanzee SNP datasets (i.e., CW25, CW10, CW5, and CdbSNP136, all of which contain intronic SNPs). We found that the coSNP_*O/E*_ value in introns was significantly lower than the value at zero-fold degenerate sites (*P* value < 0.01), but was close to the value at nonzero-fold degenerate sites (Fig. 2a). This result suggested that coSNP_*O/E*_ might be elevated at zero-fold degenerate sites, rather than being reduced at nonzero-fold degenerate sites. Overall, these trends observed in coding coSNPs between human and chimpanzee (Fig. 2a) still held true between human and non-human primates (Fig. 2b) and between non-human primates (Fig. 2c).

It is noteworthy that the human SNPs retrieved from dbSNP Build138 are more abundant and comprehensive (in terms of population size) than the total chimpanzee SNPs used in this study (963,049 vs. 152,392 coding SNPs, excluding SNPs located at CpG dinucleotides). It is therefore important to determine whether the relatively limited number of chimpanzee SNPs might introduce bias that resulted in the observed trends for coSNP_*O/E*_. To address this issue, we first retrieved human SNPs from a relatively small human SNP dataset (41,391 coding SNPs) generated using nine individuals^10^, and calculated the coSNP_*O/E*_ between these human SNPs and the total number of the chimpanzee SNPs analyzed in the study. The above-mentioned trends that (i) the values of coSNP_*O/E*_ was significantly greater than one, regardless of the level of degeneracy of coding nucleotides (all *P* values < 10^−15^), (ii) zero-fold degenerate sites had a higher coSNP_*O/E*_ than nonzero-fold degenerate ones, and (iii) the coSNP_*O/E*_ values in introns were closer to those at nonzero-fold degenerate sites than those at zero-fold ones still observed (Supplemental Fig. S2). Moreover, we estimated the number of chimpanzee coding SNPs and coSNPs using human SNPs (dbSNP138) and each of the five chimpanzee SNP datasets (i.e., CE6, CE12, CW5, CW10, and CW25 SNPs) to simulate the growth for when the number of chimpanzee individuals was very large (e.g., ≥1,000). We used linear regression model with logarithmic transformations (or log-linear model) to project the number of zero-, two-/three-, and four-fold degenerate SNPs and then coSNPs for each of chimpanzee SNP datasets, as the number of individuals approached 1,000 (METHODS; Supplemental Figs S3a and S3b). The aforementioned trends were maintained under such simulations (Supplemental Fig. S3c), suggesting that the examined population size, whether human or chimpanzee, did not change our finding.

### Sequence contexts and shared ancestral polymorphisms cannot fully account for the discrepancy of coSNP_*O/E*_ between zero- and nonzero-fold degenerate sites

**Figure 3:**
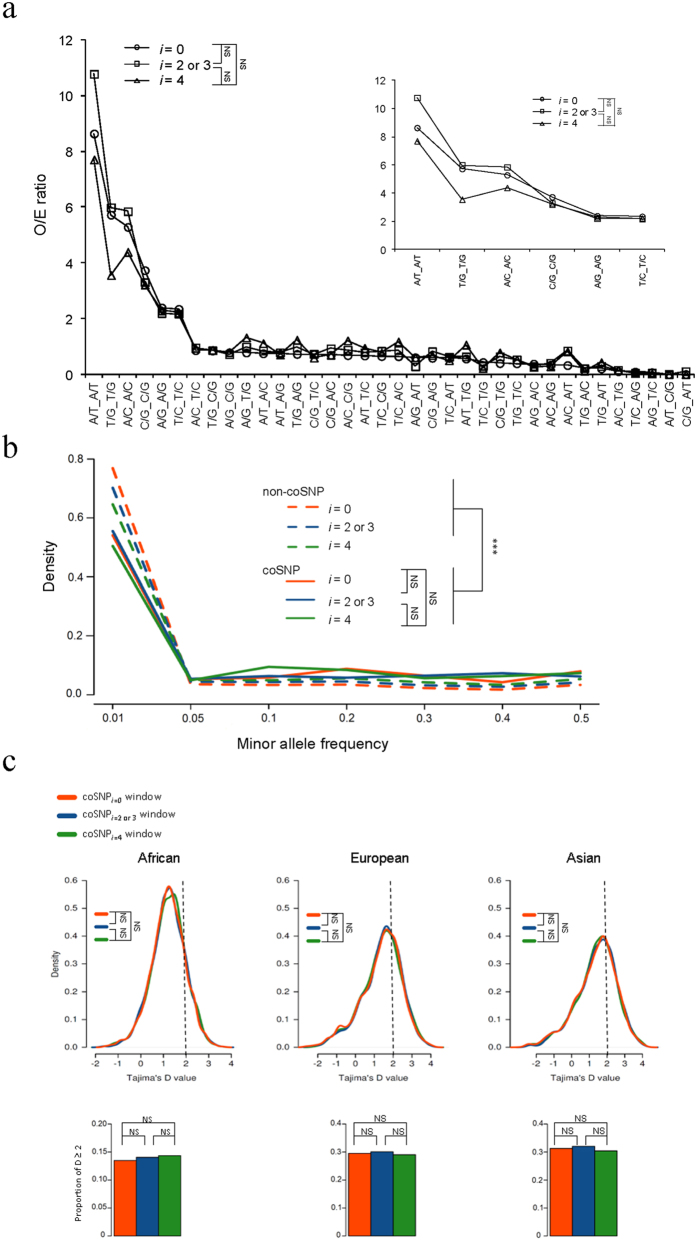
Comparisons of (**a**) the observed-to-expected (*O/E*) ratio for each type of coSNP patterns (see also Supplemental Table S4), (**b**) the SFS distributions of human-chimpanzee coSNPs and human non-coSNPs at zero-fold (*i* = 0), two-/three-fold (*i* = 2 or 3), and four-fold (*i* = 4) degenerate sites as a function of human minor allele frequency (dbSNP138), and (**c**) distributions of Tajima’s D values between coSNP_*i*=0_, coSNP_*i*=2/3_, and coSNP_*i*=4_ windows (see the text) in African, European, and Asian populations. The upper right panel of (**a**) represents the *O/E* ratios for the six types of coSNP patterns with the same two alleles in both species. Statistical significance was estimated by (**a**) the paired *t*-test and (**b**,**c**) the Kolmogorov-Smirnov test, respectively. The bottom panel for each population of (**c**) represents the proportions of Tajima’s D values ≥ 2 in coSNP_*i*=0_, coSNP_*i*=2/3_, and coSNP_*i*=4_ windows, as indicated, in which the *P* values were determined by *Chi*-square test of equal proportions. Significance: ***P < 0.001. NS, not significant.

We subsequently sought possible explanations for the elevation of coSNP_*O/E*_ at zero-fold degenerate sites. We combined the six chimpanzee SNP datasets described above, and retrieved a total of 4,375, 3,341, and 2,479 human-chimpanzee coding coSNPs at zero-, two-/three-, and four-fold degenerate sites (Table 2), respectively. Initially, we calculated the observed-to-expected ratio (*O/E* ratio) for each type of coSNP pattern, i.e., six dimorphic nucleotide patterns (e.g., A/C, A/G, A/T, C/G, C/T, and G/T) detected at the orthologous sites in both human and chimpanzee, at zero-, two-/three-, and four-fold degenerate sites (see Supplemental Table S4 and Fig. 3a). We compared the trends of coSNP patterns of the three types of *i*-fold degenerate coSNPs, and found no statistically significant difference between them (all *P* values > 0.05 by the paired *t*-test; Fig. 3a). This indicated that different types of *i*-fold degenerate coSNPs exhibited similar coSNP patterns. We then examined whether sequence contexts could account for the discrepancy of coSNP_*O/E*_ between zero-fold and nonzero-fold degenerate sites. Since certain sequence contexts/motifs might be associated with mutational hotspots^2^,^13^, we investigated whether specific motifs contributed to our finding. We examined potential composite motifs of coSNP loci and their flanking three nucleotides using Weblogo3^14^, and found that neither zero-fold nor nonzero-fold degenerate coSNPs was related to any specific motif (all entropy values were close to zero; Supplemental Fig. S4a). We also performed *de novo* motif finding in the flanking regions of coSNPs (within −50 nucleotides to +50 nucleotides of the examined sites) using MEME^15^, and found no difference of sequence motif between the flanking regions of zero-fold and nonzero-fold degenerate coSNPs (Supplemental Fig. S4b). Of note, since the observation remained true even excluding CpG dinucleotides (Fig. 2), mutagenesis at CpG dinucleotides seemed unlikely to be a major cause of this regard. Therefore, these results suggested that sequence contexts could not simply explain the discrepancy of coSNP_*O/E*_ between zero-fold and nonzero-fold degenerate sites.

After excluding sequence contexts as the cause of the discrepancy, we addressed the possibility that human-chimpanzee shared ancestral polymorphisms (whether they were maintained either by chance or by balancing selection) might account for the increase of coSNP_*O/E*_. Four lines of evidence indicated that shared ancestral polymorphisms were unlikely to account for the discrepancy of coSNP_*O/E*_ between zero- and nonzero-fold degenerate sites. First, shared ancestral polymorphisms should exhibit the same two alleles in both human and chimpanzee (e.g., a G-T SNP in human to be coincident with a G-T SNP in chimpanzee). Considering the observed-to-expected ratios for the six types of coSNP patterns with the same two alleles in both species, there was no significant difference between any two types of *i*-fold degenerate coSNPs (all *P* values > 0.05 by the paired *t*-test; the upper-right panel of Fig. 3a and Supplemental Table S4). Second, we determined the site frequency spectrum (SFS) of coding coSNPs; if zero-fold degenerate coSNPs had a higher proportion of SNPs originating from the human-chimpanzee common ancestor than nonzero-fold ones, a higher proportion of zero-fold degenerate coSNPs might have survived genetic drift in both species, which would be represented by a flatter SFS^16^. Although coSNPs generally exhibited a flatter SFS than non-coSNPs (all *P* values < 10^−15^ by the Kolmogorov-Smirnov test), which suggested that coSNPs might consist of a higher proportion of SNPs that originated from the human-chimpanzee common ancestor than non-coSNPs, there were no differences between the SFS distributions of any two types of *i*-fold degenerate coSNPs (all *P* values > 0.05; Fig. 3b). This indicated that shared ancestral polymorphisms might not be a major factor for the trend of a higher coSNP_*O/E*_ at zero-fold degenerate sites than at nonzero-fold degenerate ones. Third, we retrieved Tajima’s D values of non-overlapping 100k-bp windows on the basis of three SNP datasets from different human populations (i.e., African, European, and Asian; see METHODS), and classified the windows into three groups: (i) the windows containing four-fold degenerate coSNPs but no other types of *i*-fold degenerate coSNPs (“coSNP_*i*=4_ windows”), (ii) the windows containing two-/three-fold degenerate coSNPs but no zero-fold degenerate coSNPs (“coSNP_*i*=2or3_ windows”), and (iii) the windows containing zero-fold degenerate coSNPs (“coSNP_*i*=0_ windows”). It should be noted that the coSNP_*i*=2or3_ windows might contain four-fold degenerate coSNPs, and the coSNP_*i*=0_ windows might contain two-/three-fold and/or four-fold degenerate coSNPs. We found that the distributions of Tajima’s D values and the proportions of windows with Tajima’s D values ≥ 2 (representing the regions under balancing selection or population contraction) were no different between any two types of windows, regardless of the human population examined (all *P* values > 0.05 by the Kolmogorov-Smirnov test and *Chi*-square test of equal proportions, respectively; Fig. 3c). Finally, the trend of a higher coSNP_*O/E*_ at zero-fold degenerate sites than at nonzero-fold degenerate ones holds well between hominoid species (including human and other great apes) and rhesus macaque (Fig. 2b,c), which diverged more than 23 million years ago^17^. Preservation of higher-than-expected polymorphisms over such evolutionary time is improbable. Taken together, we thus suggested that shared ancestral polymorphisms and balancing selection cannot account for the elevated coSNP_*O/E*_ at zero-fold degenerate sites.

### Density of single SNPs and recombination rate are not the major cause of the discrepancy of coSNP_*O/E*_ between zero- and nonzero-fold degenerate sites

**Figure 4:**
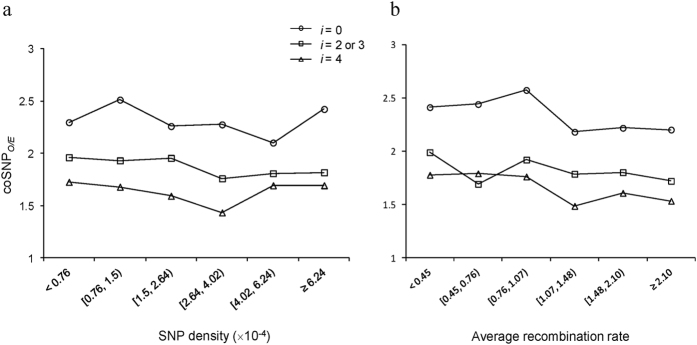
Distribution of coSNP_*O/E*_ of zero-fold (*i* = 0), two-/three-fold (*i* = 2 or 3), and four-fold (*i* = 4) degenerate nucleotides in the non-overlapping 1 M-bp windows (see the text) of different levels of (**a**) SNP density and (**b**) average recombination rate.

As coSNP density was observed to be positively correlated with the density of single SNPs (e.g., human SNPs) and recombination rate^4^ (Supplemental Fig. S5), we were curious about whether these two factors may affect the discrepancy of coSNP_*O/E*_ between zero-fold and nonzero-fold degenerate sites. We thus calculated the density of single SNPs and retrieved the average recombination rates of non-overlapping 1M-bp windows (METHODS), respectively. We classified the windows into different groups according to the single SNP density and the combination rates, respectively, and calculated the coSNP_*O/E*_ values at zero, two-/three-fold, and four-fold degenerate nucleotides for each group. Our results revealed that (i) the trend of a higher coSNP_*O/E*_ at zero-fold degenerate sites than at nonzero-fold degenerate ones remained across all groups of different single SNP densities (Fig. 4a) and different recombination rates (Fig. 4b); and (ii) coSNP_*O/E*_ were not significantly correlated with single SNP density (Fig. 4a) and recombination rate (Fig. 4b), regardless of the degeneracy of coding nucleotides (all *P* values > 0.05 by the two-tailed Spearman’s rank correlation test). These results suggested that the density of single SNPs and recombination rate were not the major cause of the elevation of coSNP_*O/E*_ at zero-fold degenerate sites.

### The effect of degeneracy of coding nucleotides on coSNP_*O/E*_ is dependent on the strength of selective constraints

**Figure 5:**
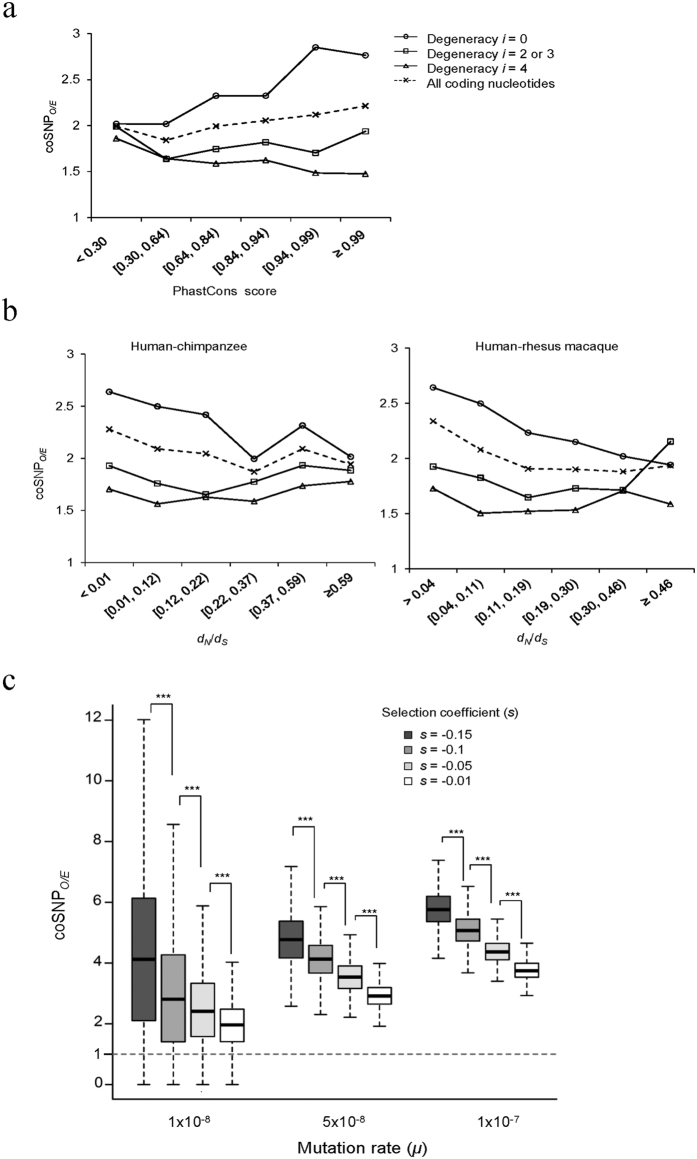
Comparisons of coSNP_*O/E*_ and the strength of selective constraints in coding regions. (**a**,**b**) Distribution of coSNP_*O/E*_ of zero-fold (*i* = 0), two-/three-fold (*i* = 2 or 3), and four-fold (*i* = 4) degenerate nucleotides in (**a**) coding exons and (**b**) protein-coding genes under different levels of selective constrains measured by PhastCons scores and *d*_*N*_*/d*_*S*_ ratios, respectively. (**c**) The effect of selective constraints and mutation rate on coSNP_*O/E*_ of coding sequences on the basis of the SLiM simulation (see the text and METHODS).

Since zero-fold degenerate sites are generally subject to stronger selective constraints than nonzero-fold degenerate sites^6^, we reasoned that the selective constraints might affect the excess of coSNPs. To address this possibility, we separated coding exons and genes into different groups of similar size according to the evolutionary rates measured by the PhastCons scores^18^ and *d*_*N*_*/d*_*S*_ (nonsynonymous to synonymous substitution rate) ratios, respectively. Our results revealed that the coSNP_*O/E*_ values of all coding nucleotides were positively correlated with the PhastCons scores for the exon level (Fig. 5a) and negatively correlated with *d*_*N*_*/d*_*S*_ ratios for the gene level (Fig. 5b), indicating a positive correlation between coSNP_*O/E*_ and the strength of selective constraints. We further calculated the coSNP_*O/E*_ values for each exon/gene group at zero-fold, two-/three-fold, and four-fold degenerate sites, respectively. In general, without respect to the exon or gene levels, we found that (i) the level of discrepancy of coSNP_*O/E*_ between zero-fold and nonzero-fold degenerate sites increased with increasing strength of selective constraints; and (ii) the strength of selective constraints was positively correlated with coSNP_*O/E*_ at zero-fold degenerate sites (all *P* values < 0.05 by the one-tailed Spearman’s rank correlation test), whereas such a trend was not observed at both two-/three-fold and four-fold degenerate nucleotides (all *P* values > 0.5) (Fig. 5a,b). These results revealed that the effect of degeneracy on coSNP_*O/E*_ was dependent on the strength of selective constraints, and purifying selection has contributed to elevated coSNP_*O/E*_ at zero-fold degenerate nucleotides, suggesting the involvement of selective constraints in shaping distribution of coSNPs in coding regions.

We then asked whether mutation rate may affect the correlation between coSNP_*O/E*_ and purifying selection. We used SLiM^19^ to simulate sequence variation under arbitrary models of selection and demography, and showed that coSNP_*O/E*_ increased significantly with increasing the strength of selective constraints, regardless of the level of mutation rate (all *P* values < 10^−5^ by the two-tailed Wilcoxon rank sum test; Fig. 5c). The two-way ANOVA analysis also revealed that the interaction of the effect of these two factors (the strength of selective constraints and mutation rate) on coSNP_*O/E*_ was not statistically significant (*P* value = 0.156; Supplemental Table S5). These results thus suggested the independence between these two factors in affecting the distribution of coSNPs.

### coSNPs tend to be less damaging than non-coSNPs at zero-fold degenerate sites

**Figure 6:**
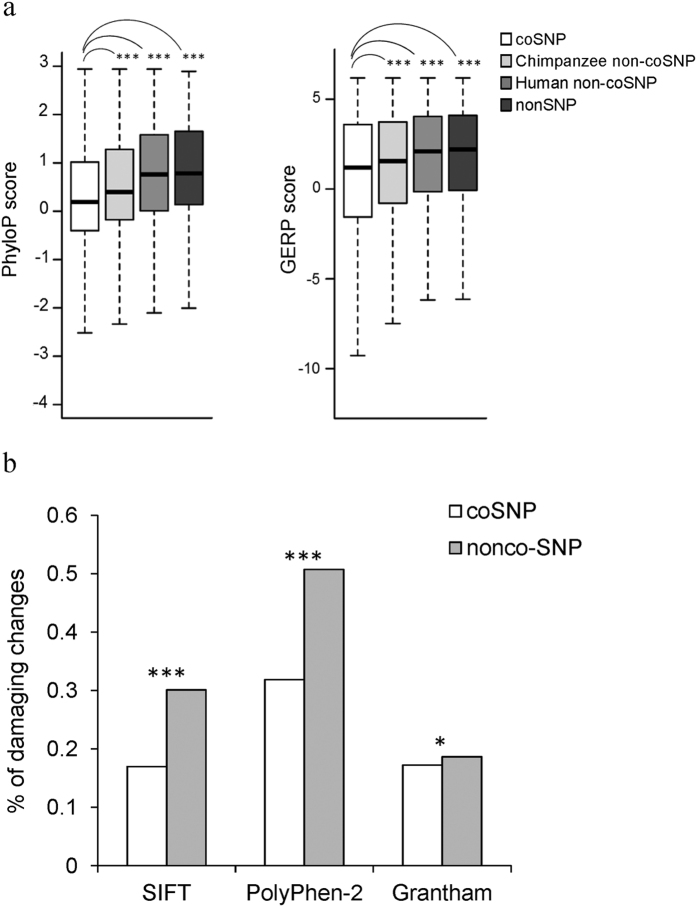
Estimation of functional consequences of nonsynonymous coSNPs. (**a**) Comparisons of the conservation scores (measured by PhyloP and GERP) of coSNPs and their nearest neighbor human/chimpanzee non-coSNPs and non-SNPs at zero-fold degenerate sites. Human (or chimpanzee) non-coSNPs represent that SNPs are observed in human (or chimpanzee) but not in chimpanzee (or human) at the human-chimpanzee orthologous sites. (**b**) Comparisons of the percentages of damaging changes of coSNPs and human non-coSNPs at zero-fold degenerate sites (measured by SIFT, PolyPhen-2, and Grantham). Statistical significance was estimated by (**a**) the two-tailed Fisher’s exact test and (**b**) the paired Wilcoxon rank-sum test, respectively. Significance: *P < 0.05, **P < 0.01, and ***P < 0.001.

We proceeded to investigate whether zero-fold degenerate sites with coSNPs are subject to more relaxed selective pressure than those with non-coSNPs, resulting in the elevated coSNP_*O/E*_ at zero-fold degenerate sites. We thus examined the conservation scores determined by PhyloP^20^ and GERP^21^ for each coSNP and its nearest neighbor human non-coSNP, chimpanzee non-coSNP, and nonSNP at zero-fold degenerate sites within the same gene. Figure 6a showed that coSNPs exhibited a significantly lower level of conservation than both non-coSNPs and nonSNPs at zero-fold degenerate sites (all *P* values < 10^−15^ by the paired *t*-test), suggesting that at zero-fold degenerate sites coSNPs might be under more relaxed selection pressure than their neighbor non-coSNPs and nonSNPs. We further examined the proportions of damaging changes, which were measured by SIFT^22^, PolyPhen-2^23^, and Grantham^24^, for coSNPs and non-coSNPs (human) at zero-fold degenerate sites. All the three predictions suggested that at zero-fold degenerate sites coSNPs had significantly lower proportions of damaging changes than non-coSNPs (all *P* values < 0.05 by the two-tailed Fisher’s exact test; Fig. 6b). These results echoed our previous observation that coSNPs had a significantly lower proportion of rare variants (minor allele frequency < 1%) than non-coSNPs (0.53 vs. 0.75, *P* value < 10^−15^ by the two-tailed Fisher’s exact test) at zero-fold degenerate sites (Fig. 3b). Generally, common SNPs (i.e., minor allele frequency ≥ 1%) might be under weaker selective constraints than rare SNPs^25^. Zero-fold degenerate coSNPs had a high proportion of common SNPs, also supporting that they tended to be tolerant.

### Zero-fold degenerate coSNPs are associated with human diseases

**Figure 7:**
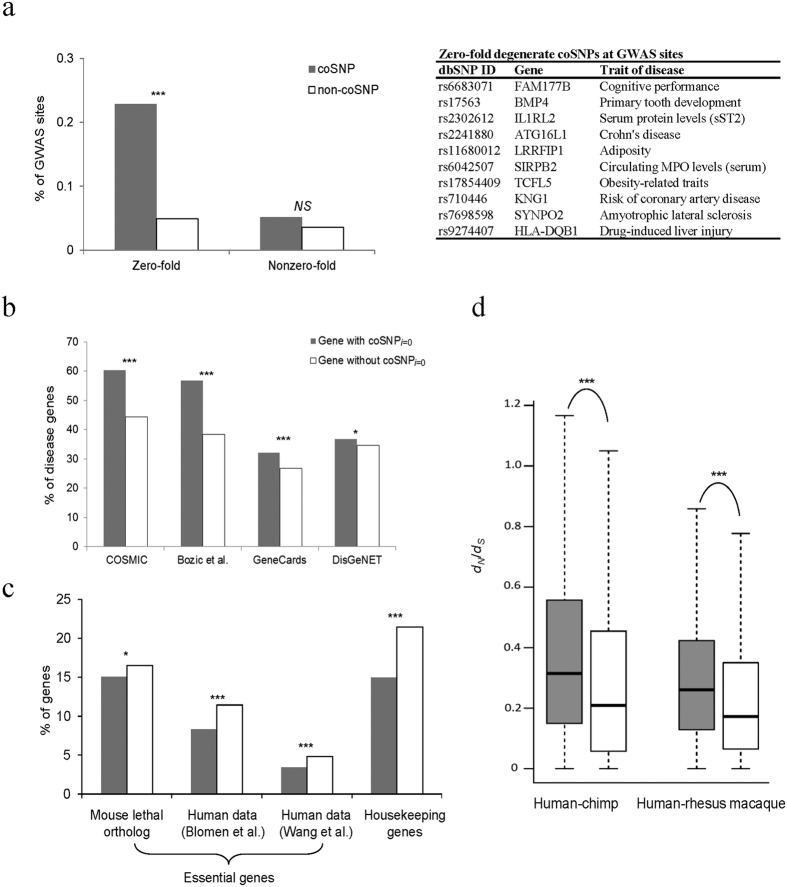
Functional analysis of the zero-fold degenerate coSNPs and the genes containing zero-fold degenerate coSNPs (genes with coSNP_*i*=0_). (**a**) The percentages of disease-associated SNPs (GWAS sites) of coSNPs and non-coSNPs at zero- and nonzero-fold degenerate nucleotides (left part), and the list of the zero-fold degenerate coSNPs at GWAS sites (right part). MPO: myeloperoxidase. (**b**,**c**) The percentages of (**b**) disease-associated genes (extracted from the four databases/studies: COSMIC, that of Bozic *et al*., GeneCards, and DisGeNET) and (**c**) essential/housekeeping genes of genes with/without coSNP_*i*=0_. (**d**) Comparison of *d*_*N*_*/d*_*S*_ ratios of genes with and without coSNP_*i*=0_. Statistical significance was estimated by (**a**–**c**) the two-tailed Fisher’s exact test and (**d**) the two-tailed Wilcoxon rank-sum test, respectively. Significance: *P < 0.05 and ***P < 0.001. NS, not significant.

We further examined the association between zero-fold degenerate coSNPs and human diseases. First, on the basis of information about disease-associated SNPs, i.e., the associations identified in the genome-wide association studies (GWAS), we found that at zero-fold degenerate sites coSNPs had a significantly higher percentage of GWAS sites than non-coSNPs (*P* value < 10^−4^ by the two-tailed Fisher’s exact test), whereas such a trend was not observed at nonzero-fold degenerate ones (*P* value = 0.47) (Fig. 7a). Second, we examined the association between genes containing zero-fold degenerate coSNPs (genes with coSNP_*i*=0_; 3,106 genes) and human disease genes. We extracted disease-associated genes from four well-known datasets: that of Bozic *et al*.^26^, COSMIC^27^, GeneCards^28^, and DisGeNET^29^. We found that genes with coSNP_*i*=0_ had a significantly higher proportion of human disease genes than the other genes (i.e., genes without coSNP_*i*=0_; 14,076 genes) (all *P* values < 0.05, Fig. 7b). These results thus suggested that zero-fold degenerate coSNPs were associated with human diseases at either nucleotide or gene level.

Intriguingly, we found that genes with coSNP_*i*=0_ were depleted in essential (including human orthologues of mouse lethal genes^30^,^31^ and human essential genes^32^,^33^) and housekeeping genes as compared to those without coSNP_*i*=0_ (all *P* values < 0.05, Fig. 7c). Meanwhile, genes with coSNP_*i*=0_ had significantly lower *d*_*N*_/*d*_*S*_ values than those without coSNP_*i*=0_ for either human-chimpanzee or human-rhesus macaque orthologues (both *P* values < 0^−15^ by the two-tailed Wilcoxon rank sum test, Fig. 7d), suggesting that the former were subject to weaker selective constraints than the latter. This also reflected a previous observation that disease genes tended to be less evolutionary conserved than essential/housekeeping genes^34^. Furthermore, by performing DAVID^35^,^36^ for the gene enrichment analysis, we found that genes with coSNP_*i*=0_ were enriched in olfaction- and cell membrane-related categories (Supplemental Table S6). Olfaction-related genes are known to be subject to relaxed selection pressure, because of the diminishing importance of olfaction during human evolution^37^,^38^. Meanwhile, cell membrane-related genes have a general disposition of containing long intrinsically disordered regions^39^,^40^,^41^, which have been suggested to evolve faster than ordered regions^42^,^43^,^44^. These results also supported the above observation that genes with coSNP_*i*=0_ were under more relaxed selection pressure than those without coSNP_*i*=0_ (Fig. 7d).

## Discussion

To the best of our knowledge, this is the first study to globally investigate coincident SNPs in primate protein-coding regions. We first sequenced the exomes of six unrelated chimpanzees, and then identified their coding SNPs. We found that 86% (9,615) of the identified coding SNPs were novel to the chimpanzee dbSNP (Build 136), and that 29% (3,249) of them were previously uncharacterized in the published chimpanzee SNP datasets (CdbSNP, CE12, CW5, CW10, and CW25 SNPs). The newly identified SNPs may enhance our knowledge of genetic variations between chimpanzees. Next, we pinpointed human-chimpanzee coSNPs by comparing human SNPs with the six chimpanzee SNP datasets, and showed that coding regions, just as whole genome, contain an excess of coSNPs. Intriguingly, we showed that zero-fold degenerate sites had a greater enrichment of coSNPs (based on coSNP_*O/E*_) than nonzero-fold degenerate sites, and such a difference was due to an elevation of coSNP_*O/E*_ at zero-fold degenerate sites, rather than a reduction at nonzero-fold degenerate sites. These tendencies were independent of chimpanzee subpopulation, examined population size, sequencing protocol, or sequencing platform, and generally held true between primates, even for hominoid-rhesus macaque coSNPs.

**Figure 8:**
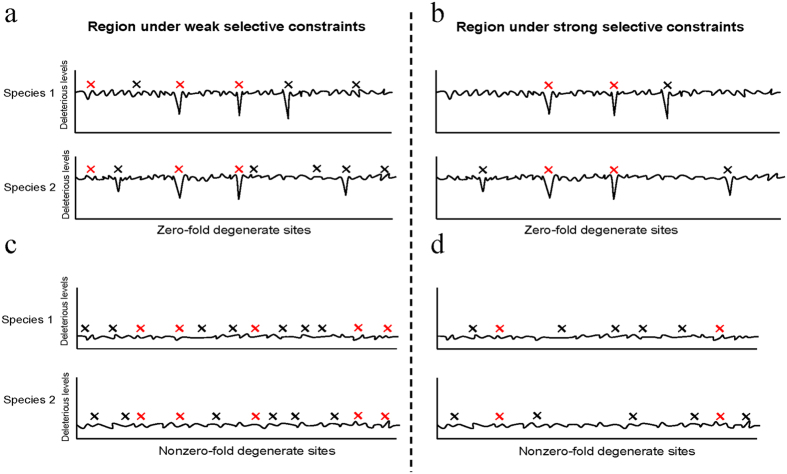
A schematic diagram for SNP distributions of zero-fold degenerate sites in a region under (**a**) weak and (**b**) strong selective constraints and nonzero-fold degenerate sites in a region under (**c**) weak and (**d**) strong selective constraints. Black and red crosses represent single SNPs and coSNPs, respectively.

To investigate the reason of the differences in coSNP_*O/E*_ between zero-fold and nonzero-fold degenerate sites, we established that none of sequence contexts, shared ancestral polymorphisms, density of single SNPs, and recombination rate was the major causes. We demonstrated that (i) the strength of selective constraints remarkably affected the level of discrepancy of coSNP_*O/E*_ between zero-fold and nonzero-fold degenerate sites (Fig. 5a,b), (ii) the strength of selective constraints was positively correlated with coSNP_*O/E*_ at zero-fold degenerate sites, whereas such a trend was not observed at nonzero-fold degenerate ones (Fig. 5a,b), and (iii) selection and mutation rate affected coSNP_*O/E*_ independently in coding sequences (Fig. 5c). We further showed that coSNPs tended to be less damaging than non-coSNPs at zero-fold degenerate sites, and that the zero-fold degenerate sites with coSNP tended to be more tolerant of mutations and under more relaxed selection pressure than those with non-coSNPs and nonSNPs (Fig. 6). These observations all pointed to the conclusion that the elevated coSNP_*O/E*_ at zero-fold degenerate sites is associated with selection pressure. It is known that zero-fold degenerate sites are generally under stronger selective constraints than nonzero-fold degenerate sites, resulting in the selective elimination of the majority of zero-fold degenerate SNPs^6^. If a region is under stringent selective constraints, most zero-fold degenerate SNPs are selectively eliminated except for the zero-fold degenerate sites that are relatively tolerant of mutations (Fig. 8a,b). As such, the observed zero-fold degenerate SNPs were more frequent to be coSNPs (resulting in a higher coSNP_*O/E*_) in the regions under stringent selective constrains than in those under relaxed selection pressure (Fig. 8a,b). In contrast, nonzero-fold degenerate sites (particularly four-fold degenerate sites) generally had a higher tolerance of mutations than zero-fold degenerate ones, and thus SNPs at nonzero-fold degenerate sites tended to be homogenized, regardless of strength of selective constraints (Fig. 8c,d). Therefore, the trend of a higher coSNP_*O/E*_ at zero-fold than at nonzero-fold degenerate sites was relatively significant in the regions that were subject to stringent selective constrains. Taken together, our study suggested that purifying selection was important in shaping the distribution of coSNPs in primate coding regions.

Functional analysis further revealed that coSNPs had a significantly higher percentage of disease-associated SNPs (i.e., GWAS sites) than non-coSNPs at zero-fold degenerate sites (Fig. 7a), and genes with coSNP_*i*=0_ were enriched in human diseases as compared with those without coSNP_*i*=0_ (Fig. 7b). These results suggested that zero-fold degenerate coSNPs were associated with human diseases, implying that the orthologous polymorphisms of these human disease-associated SNPs might also be associated with the corresponding diseases in the compared species. A prominent example is rs2241880. This SNP encoding a missense variant in *ATG16L1* is strongly associated with Crohn’s disease (a chronic inflammatory bowel disease) among human populations, and its orthologous polymorphism also results in similar intestinal inflammation in mouse^45^. Interestingly, rs2241880 is also a human-chimpanzee coSNP (Fig. 7a, right). Whether its orthologous polymorphism is also associated with similar diseases in chimpanzee awaits further investigation. In addition, we found that gene with coSNP_*i*=0_ were enriched in the functional categories of cognition and neurological system process (Supplemental Table S6), and two zero-fold degenerate coSNPs at GWAS sites, rs6683071 and rs7698598 (Fig. 7a, right), which were demonstrated to connect to cognitive performance^46^ and amyotrophic lateral sclerosis^47^, respectively. Therefore, whether zero-fold degenerate coSNPs have contributed to human-chimpanzee divergences in the cognition and neurological system is worthy of further investigation. On the other hand, the gene enrichment analysis also showed that gene with coSNP_*i*=0_ were enriched in genes related to glycoproteins (Supplemental Table S6). It was reported that haplotypes and coding polymorphisms shared by human and chimpanzee were enriched in membrane glycoproteins, which were likely to be maintained by balancing selection^48^. Consequently, although balancing selection might not be the most likely explanation for the majority of coSNPs^3^ and the discrepancy of coSNP_*O/E*_ between zero-fold and nonzero-fold degenerate sites (this study), some of the observed coSNPs might be subject to balancing selection. In addition, although the strength of selective constraints and mutation rate affect the distribution of coSNPs independently (Fig. 5c and Supplemental Table S5), our simulation result also showed that the prevalence of coSNPs was associated with the level of mutation rate (Fig. 5c), suggesting that highly mutable regions (e.g., disease-associated genes^49^) were more likely to become substrates of coSNPs.

Rather than performing study on a genome-wide scale, this study focuses on the coSNPs located in coding regions, and thus offers a deeper analysis of coSNPs at a finer resolution than described previously. The conclusion that the distribution of coding coSNPs is dependent on the degeneracy of coding nucleotides and the strength of selective constraints further implies that coSNPs may represent an evolutionary “signature” of coding sequences, thus providing new insights into the context of evolutionary biology.

## Methods

### Blood sampling and exome sequencing

Whole blood cells for genomic DNA extraction were obtained from six unrelated chimpanzees (Supplemental Table S1) housed at Taipei Zoo, Taiwan. All samples were approved by the Council of Agriculture Executive Yuan, Taiwan (Approval number: 0961701136). The methods were carried out in accordance with the approved guidelines. Genomic DNA was isolated using the Genomic DNA mini Kit (Geneaid, Taiwan), and then stored at −80 °C. The SureSelect^TM^ Human All Exon Kit, 38 Mb (Agilent Technologies, Santa Clara, CA, USA; including all unique well-annotated protein-coding regions from the CCDS database (March 2009)^8^) was used to capture the exome of each chimpanzee. Of note, the SureSelect^TM^ Human All Exon Kit has been successfully applied to capturing genomic DNA of non-human primates such as chimpanzee and rhesus macaque^9^,^50^. The captured regions included the 10 bp regions flanking the targeted exons (a total of 29,516,842 bp). All samples from the six chimpanzees were sequenced on the massively parallel sequencer SOLiD^TM^ 4 System, using the 50-bp single-read protocol. All samples were run in 2 wells, except for sample 20050256B10, which was run in 4 wells (Supplemental Table S1). Sample 20050256B10 was also sequenced using the SOLiD^TM^ 3 Plus System.

### Read mapping and SNP calling

The human (hg19) and chimpanzee (panTro3) reference genomic sequences were downloaded from the UCSC genome browser. For each sample, the SOLiD reads were aligned against the chimpanzee reference genome using Novoalign (v 2.7.17) (Novocraft Technologies) with default parameters (parameters of gap penalty: (-g 40 -x 6)). Only the uniquely matched reads mapped on the human-chimpanzee orthologous consensus coding sequences (CCDSs)^8^ were considered. Human-chimpanzee orthologous CCDSs were determined using the LiftOver tool^51^, on the basis of human-chimpanzee pairwise alignments (downloaded from the UCSC genome browser) which included 155,276 coding exons and their flanking 10 bases (a total of 29,516,842 bases). We found that 53.7% ~ 64.8% of reads were uniquely mapped on the chimpanzee genome, and the average coverage depth was greater than 45-fold for all six exomes (Supplemental Table S1). When considering the targeted regions (29.5 Mb in length), including the human-chimpanzee orthologous CCDSs and their flanking 10-base regions, >90% of targeted bases were covered at least once, and >80% were covered sufficiently for variant calling (≥8× coverage) (Supplemental Table S2). To minimize possible mapping errors, mapped regions with low read coverage (<8× coverage) and regions located within CNVs^52^ or repetitive regions (defined by RepeatMasker; downloaded from the UCSC genome browser) were excluded. We also mapped the reads generated by the SOLiD 3 Plus System to the chimpanzee reference genome, revealing a similar unique mapping rate level (56.4%), coverage depth (49.7-fold) (Supplemental Table S1), and target coverage (≥8×; 75.5%). The read depth of SOLiD-3-Plus data was also highly correlated with that of SOLiD-4 data (*r* = 0.954, *P* value < 10^−15^ by the Pearson’s correlation test; Supplemental Fig. S6). These results indicate the stability of our mapping statistics. Ultimately, 20,895,577 bases were retained.

SNPs were called from the retained sequences using SAMtools (v 0.1.18)^53^ with a call quality value (QV) ≥ 30. We excluded bases with a sequence quality score <20 and reads with multiple genetic variants. The accuracy of the called variants was further improved by considering only the called SNPs that satisfied all of the following criteria: (1) of the six chimpanzee individuals, there must be at least one homozygous individual in which both alleles are the same as the chimpanzee reference genome, to minimize false positives arising from possible errors in the chimpanzee reference genome; (2) they must be simultaneously supported by the left- and right-half parts of reads, to eliminate potential mapping errors (examples are given in Supplemental Fig. S7); and (3) they must also be identified by SAMtools on the basis of the Novoalign alignments with non-default parameters of gap penalty (e.g., (-g 100 -x 5)).

Three lines of evidence indicated that our results were unlikely to be a consequence of bias in the selection of sequencing techniques. First, the transition-to-transversion (Ts/Tv) ratio of the identified chimpanzee SNPs was 2.7, which was similar to that obtained from human exome SNP calling (2.7~3.5). Second, since nonsynonymous SNPs were most likely to be deleterious, they tended to have a low derived-allele frequency within a population. We found that the derived allele frequency distribution (inferred from the human reference genome) of the identified nonsynonymous SNPs exhibited a high proportion of low-frequency derived alleles (Supplemental Fig. S8). Third, the chimpanzee SNP datasets analyzed in this study consisted of different sequencing protocols (exome and whole-genome sequencing) and different sequencing platforms (SOLiD and Illumina sequencers) (Table 1). The observed tendencies were independent of sequencing protocol and sequencing pla3tform (see Figs 1a and 2a).

### Collection of primate SNP datasets

The human (dbSNP138) and chimpanzee (dbSNP136) SNP datasets were downloaded from the NCBI FTP server at ftp://ftp.ncbi.nih.gov/snp/organisms/. The other chimpanzee SNP datasets used in this study (i.e., CE12, CW5, CW10, and CW25 SNPs) were summarized in Table 1. The gorilla SNPs were obtained from an earlier study^10^. The orangutan SNPs were obtained from dbSNP136 (NCBI) and two earlier studies^10^,^12^. The rhesus macaque SNPs were collected from dbSNP136 (NCBI) and an earlier study^12^. The gorilla, orangutan, and rhesus macaque SNPs used in this study are summarized in Supplemental Table S3. Human SNPs from a small population (nine individuals) were obtained from an earlier study^10^. The coordinates of the non-human primate SNPs were converted to their human orthologous positions (hg19) using the LiftOver tool, on the basis of the UCSC alignments.

### Data retrieval and availability

The human gene annotation was downloaded from the Ensembl genome browser (Release 73) at http://www.ensembl.org/index.html. Degeneracy of coding nucleotides was determined on the basis of the Ensembl gene annotation, in which nucleotides with ambiguous degeneracy (e.g., caused by overlapping genes or alternative splicing) were not considered. The motif analysis of coSNP loci and their flanking regions were evaluated using Weblogo3^14^ and MEME^15^, respectively. The Weblogo3 analysis was performed on the Galaxy web-based platform. The MEME tool was downloaded from the MEME Suite at http://meme-suite.org/. The Tajima’s D values of non-overlapping 100k-bp windows derived from the SNPs of three human populations (African, European, and Asian)^54^ and the average recombination rates of non-overlapping 1M-bp windows based on the deCODE genetic map^55^ were both downloaded from the UCSC genome browser at http://genomes.ucsc.edu/. The PhyloP^20^ and GERP^21^ scores were used to measure the conservation levels of single nucleotides. The PhastCons scores^18^ were used to measure the conservation levels of exonic region. All these three types of scores were also downloaded from the UCSC genome browser. The evolutionary rates (*d*_*N*_/*d*_*S*_ ratios) of human-chimpanzee and human-rhesus macaque orthologous genes were downloaded from the Ensembl genome browser (Release 73). The functional consequences of variants at zero-fold degenerate sites (the variants must be nonsynonymous) were evaluated using the SIFT^56^, PolyPhen-2^23^ and Grantham^24^ scores, which were queried through the Galaxy platform at https://main.g2.bx.psu.edu/ (last accessed August 15th, 2015), the PolyPhen server (version 2.2.2) at http://genetics.bwh.harvard.edu/pph2/, and the SeattleSeq Annotation server at http://snp.gs.washington.edu/SeattleSeqAnnotation138/, respectively. In this study, “possibly” and “probably” damaging mutations were both regarded as “damaging substitutions” in the PolyPhen-2 prediction. The SIFT scores ≤0.05^56^ and the Grantham scores >100^24^ were regarded as “damaging substitutions”, respectively. The disease-associated SNPs were downloaded from GWAS at https://www.ebi.ac.uk/gwas/docs/downloads on August 4th, 2015. The human disease genes were downloaded from the four studies/databases: that of Bozic *et al*.^26^, COSMIC^27^, GeneCards^28^, and DisGeNET^29^. For the DisGeNET database, we considered the curated gene-disease associations only. The analysis of gene essentiality was performed on the basis of human orthologues of mouse lethal genes^30^,^31^ and two human essential gene sets^32^,^33^. The two human essential gene sets of Blomen *et al*.^32^ and Wang *et al*.^33^ were identified on the basis of the bacterial clustered regularly interspaced short palindromic repeats (CRISPR) system and extensive mutagenesis in haploid human cells, respectively. We only considered the “core essentialome”^32^ and the identified essential genes with *P* values < 0.05 across all examined cell lines for the gene sets of Blomen *et al*. and Wang *et al*., respectively. The human housekeeping genes were downloaded at http://www.tau.ac.il/~elieis/HKG/ 57. The gene enrichment analysis was conducted using the DAVID tools^58^,^59^.

The exome sequence data generated by this study have been deposited into the National Center Biotechnology Information (NCBI) Sequence Read Archive, under accession number SRP028744. The identified CE6 SNPs (Dataset 1), the identified coSNPs between primates (Datasets 2 and 3), the genes with human-chimpanzee coSNPs (Dataset 4), and gene information (i.e., human disease association, gene essentiality, and housekeeping) of the genes that contain zero-fold degenerate coSNPs (Dataset 5) are all publically available at http://treeslab1.genomics.sinica.edu.tw/coSNP/.

### Measurement of coSNP_*O/E*_

The ratio of observed-to-expected coSNPs (coSNP_*O/E*_) was defined as:


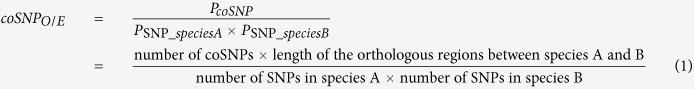


where *P*_*coSNP*_, *P*_*SNP_speciesA*_, and *P*_*SNP_speciesB*_ represent the frequencies of coSNPs, SNPs in species A, and SNPs in species B in the examined orthologous regions of the two compared species, respectively.

### Estimation of the number of chimpanzee coding SNPs and human-chimpanzee coSNPs with a chimpanzee SNP dataset of a specific number of individuals

To examine whether the observed trends in coSNP_*O/E*_ were influenced by limited numbers of chimpanzee SNPs, we estimated coding coSNPs between human SNPs (dbSNP138) and chimpanzee SNPs from each of the five chimpanzee SNP datasets (CE6, CE12, CW5, CW10, and CW25 SNPs) with a large number of individuals (e.g., 1,000). Here, we used the CE6 SNP dataset (comprised of SNPs from six chimpanzee individuals) as an example to describe the simulation process. First, as shown in Supplemental Fig. S3a, we randomly selected two of the six individuals, and calculated the numbers of chimpanzee coding SNPs and human-chimpanzee coSNPs on the basis of these two chimpanzee individuals. We then repeated the same process five times, and averaged the numbers of chimpanzee coding SNPs and human-chimpanzee coSNPs, respectively. This process was repeated by adding one individual each time, until all individuals of the CE6 SNP dataset (i.e., six individuals) were included (Supplemental Fig. S3a). Second, we used the linear regression model with logarithmic transformations (or a log-linear model) to fit the observed numbers of the chimpanzee coding SNPs and human-chimpanzee coSNPs, respectively (Supplemental Fig. S3a). Finally, we used the fitted log-linear models to estimate the numbers of chimpanzee coding SNPs and human-chimpanzee coSNPs (Supplemental Fig. S3b) and then calculated the coSNP_*O/E*_ ratio (Supplemental Fig. S3c) when the number of chimpanzee individuals was 1,000.

### Estimation of the SNPs and coSNPs in coding regions of two compared populations with different levels of selective constraints and mutation rate

To examine the effect of selective constraints and mutation rate on coSNP_*O/E*_ in coding regions, we used SLiM^19^, a forward population genetic simulator, to simulate sequence variation under arbitrary models of selection and demography. Twelve scenarios were simulated with the combinations of four levels of selective constraints (selection coefficient *s* = −0.01, −0.05, −0.1, and −0.15) and three levels of mutation rate (*μ* = 10^−8^, 5 × 10^−8^, and 10^−7^). We simulated each scenario with the parameters of the targeted region of length = 2.5 M bp and recombination rate *r* = 10^−8^ (default value). Two compared populations (with population size N = 10^4^ for each population) were then simulated 1,000 generations. After that, we calculated coSNP_*O/E*_ of the two populations on the basis of the simulated polymorphisms. Such a process was iterated 1,000 times for each scenario.

## Additional Information

**How to cite this article**: Chen, C.-Y. *et al*. Purifying selection shapes the coincident SNP distribution of primate coding sequences. *Sci. Rep.* **6**, 27272; doi: 10.1038/srep27272 (2016).

## Supplementary Material

Supplementary Information

## Figures and Tables

**Table 1:** Summary of six chimpanzee SNP datasets used in this study.

Dataset	Description (ref.)	Sequencing protocol (type of sequencer)	No. of coding SNPs
CdbSNP136	NCBI dbSNP Build 136		8,929
CE6	6 unrelated western chimpanzees (this study)	Exome (50-bp SOLiD single-end)	11,171
CE12	12 unrelated central chimpanzees^9^	Exome (90-bp Illumina paired-end)	55,063
CW5	5 unrelated chimpanzees^12^	Whole genome (101-bp Illumina paired-end)	41,788
CW10	10 unrelated western chimpanzees^11^	Whole genome (50-bp Illumina paired-end)	30,227
CW25	25 chimpanzees from Nigeria-Cameroon, Eastern, Central, and Western^10^	Whole genome (101-bp Illumina paired-end)	159,503

**Table 2:** Summary of the coSNPs between human SNPs (dbSNP138) and each of the six chimpanzee SNP datasets at zero-fold (*i* = 0), two-/three-fold (*i* = 2 or 3), and four-fold (*i* = 4) degenerate sites.

Dataset	zero-fold (*i* = 0)	two-/three-fold (*i* = 2 or 3)	four-fold (*i* = 4)
CdbSNP	190	142	112
CE6	215	172	131
CE12	1,105	1,022	722
CW5	1,012	682	506
CW10	612	463	353
CW25	2,798	2,187	1,649
**Total**	**4,375**	**3,341**	**2,479**

SNPs located within CpG dinucleotides were excluded.
