# Supplementary material for: Purifying selection shapes the coincident SNP distribution of primate coding sequences
Source: Sci Rep. 2016 Jun 3;6:27272. doi: 10.1038/srep27272 (PMC4891680; doi:10.1038/srep27272)
Supplement: Supplementary Information [file srep27272-s1.doc]

**Purifying selection shapes the coincident SNP distribution of primate coding sequences**

**Chia-Ying Chen^1^, Li-Yuan Hung^1^, Chan-Shuo Wu^1^, and Trees-Juen Chuang^1,*^**

^1^Genomics Research Center, Academia Sinica, Taipei 11529, Taiwan

***Corresponding Author**

Trees-Juen Chuang, Ph.D.

Genomics Research Center

Academia Sinica

Taipei 11529

Taiwan

E-mail: trees@gate.sinica.edu.tw

Tel: +886 2 27871244

Fax: +886 2 27899923

**Supplemental Table S1.** Summary of SOLiD sequencing reads for each chimpanzee individual.

| **Individual ID** | **Sex** | **NGS platform** | **Number of wells** | **Number of total reads** | **Number of mapped reads** | **Number of uniquely mapped reads** | **Coverage depth of analyzed reads** |
| --- | --- | --- | --- | --- | --- | --- | --- |
| 20050256B10^*^ | male | SOLiD 4 | 4 | 171,146,830 | 109,019,594  (63.70%) | 105,965,672  (61.92%) | 100.37 |
|  |  | SOLiD 3+ | 2 | 78,480,998 | 45,451,670  (57.91%) | 44,225,477  (56.35%) | 49.69 |
| 20040256B10 | male | SOLiD 4 | 2 | 102,904,080 | 67,213,850  (65.32%) | 65,060,227  (63.22%) | 55.08 |
| 20060308B10 | male | SOLiD 4 | 2 | 103,568,155 | 69,288,535  (66.90%) | 67,145,295  (64.83%) | 45.89 |
| 20060199B10 | female | SOLiD 4 | 2 | 105,797,061 | 59,030,081  (55.80%) | 56,788,418  (53.68%) | 56.16 |
| 20060235B10 | female | SOLiD 4 | 2 | 106,354,100 | 60,386,029  (56.78%) | 58,404,216  (54.91%) | 60.38 |
| 20060387B10 | female | SOLiD 4 | 2 | 103,364,130 | 65,869,658  (63.73%) | 63,643,894  (61.57%) | 50.76 |

^*^The sequencing was technically repeated on both the SOLID 4 and SOLiD 3+ platforms.

**Supplemental Table S2.** Sequence coverage across six chimpanzee exomes.

| **Individual ID** | **Covered length (bp); coverage percentage^*^** | | |
| --- | --- | --- | --- |
|  | **≥ 1X** | **≥ 5X** | **≥ 8X** |
| 20050256B10 | 27,705,434; 93.86% | 26,884,629; 91.08% | 26,380,633; 89.37% |
| 20040256B10 | 27,169,921; 92.05% | 25,709,407; 87.10% | 24,666,645; 83.57% |
| 20060308B10 | 27.125,941; 91.90% | 25,415,754; 86.11% | 24,151,701; 81.82% |
| 20060199B10 | 27,135,512; 91.93% | 25,741,636; 87.21% | 24,748,050; 83.84% |
| 20060253B10 | 27,135,654; 91.93% | 25,857,042; 87.60% | 24,955,018; 84.55% |
| 20060387B10 | 26,810,651; 90.83% | 24,983,981; 84.64% | 23,743,209; 80.44% |

**^*^**The coverage percentage = coverage length×100/length of the captured exome region. The captured exome region is 29,516,842 bp in length.

**Supplemental Table S3.** The SNP datasets of gorilla, orangutan, and rhesus macaque used in this study.

| **Primate species** | **Description (Ref)** | **No. of coding SNPs examined** |
| --- | --- | --- |
| Gorilla | - 31 individuals from Rwanda, Cameroon, and Congo[^1^](#_ENREF_1) | 105,394 |
| Orangutan | - dbSNP136 (www.ncbi.nlm.nih.gov/projects/SNP/) - 5 Sumatran and 5 Bornean individuals[^1^](#_ENREF_1) - 5 Sumatran individuals[^2^](#_ENREF_2) | 139,797 |
| Rhesus macaque | - dbSNP136 (www.ncbi.nlm.nih.gov/projects/SNP/) - 5 individuals[^2^](#_ENREF_2) | 82,752 |

**Supplemental Table S4.** The patterns of coSNPs and the observed-to-expected ratio for each type of coSNP patterns at zero-fold (*i*=0), two-/three-fold (*i*=2 or 3), and four-fold (*i*=4) degenerate sites (related to Fig. 3A).

|  | **Human SNP** | | | | | |
| --- | --- | --- | --- | --- | --- | --- |
| **Chimpanzee SNP** | **C-G** | **T-G** | **T-C** | **A-G** | **A-C** | **A-T** |
| **Observed (*i* = 0/2 or 3/4)** | | |  |  |  |  |
| **C-G** | 222/99/ 84 | 43/19/24 | 112/87/73 | 137/74/71 | 38/20/29 | 0/0/0 |
| **T-G** | 16/11/11 | 199/77/53 | 51/18/11 | 93/59/64 | 4/2/2 | 14/6/11 |
| **T-C** | 106/84/36 | 49/45/34 | 1011/872/485 | 29/19/4 | 120/84/52 | 61/49/42 |
| **A-G** | 83/96/40 | 88/83/76 | 36/19/7 | 1001/884/460 | 45/36/15 | 64/50/30 |
| **A-C** | 30/18/11 | 8/3/2 | 81/59/67 | 48/19/14 | 216/87/64 | 20/9/13 |
| **A-T** | 0/1/0 | 4/2/3 | 39/20/12 | 41/9/14 | 7/6/5 | 123/55/38 |
|  |  |  |  |  |  |  |
| **Observed/expected (*i* = 0/2 or 3/4)** | | |  |  |  |  |
| **C-G** | 3.7/3.3/3.2 | 0.84/0.85/0.87 | 0.64/0.83/0.78 | 0.78/0.72/0.79 | 0.68/0.88/1.21 | 0/0/0 |
| **T-G** | 0.39/0.63/0.78 | 5.72/5.99/3.55 | 0.43/0.30/0.22 | 0.78/0.99/1.31 | 0.10/0.15/0.15 | 0.56/0.63/1.06 |
| **T-C** | 0.71/0.72/0.59 | 0.39/0.53/0.53 | 2.34/2.17/2.22 | 0.07/0.05/0.02 | 0.86/0.96/0.93 | 0.67/0.77/0.94 |
| **A-G** | 0.58/0.82/0.68 | 0.72/0.96/1.23 | 0.09/0.05/0.03 | 2.39/2.19/2.29 | 0.33/0.40/0.28 | 0.73/0.78/0.70 |
| **A-C** | 0.69/0.92/0.69 | 0.22/0.21/0.12 | 0.64/0.87/1.17 | 0.37/0.28/0.26 | 5.28/5.85/4.39 | 0.75/0.84/1.11 |
| **A-T** | 0/0.11/0 | 0.20/0.29/0.42 | 0.58/0.62/0.50 | 0.60/0.28/0.61 | 0.32/0.85/0.81 | 8.64/10.78/7.71 |

Note. The expected frequency and number of each coSNP pattern at *i*-fold degenerate sites were estimated on the basis of the corresponding 6×6 contingency table. The six types of coSNP patterns with the same two alleles in both species were colored with gray background.

**Supplemental Table S5.** The two-way ANOVA analysis of the effect of the two factors (strength of selective constraints and mutation rate) on coSNP*_O/E_*.

| Source of Variation | df | Sum of squares | Mean square | *F* value | *P* value |
| --- | --- | --- | --- | --- | --- |
| Selection (*s*) | 3 | 6183 | 2061 | 1335.368 | < 2×10^-16^ |
| Mutation (*μ*) | 2 | 6834 | 3417 | 2213.951 | < 2×10^-16^ |
| Selection × Mutation (*sμ*) | 6 | 14 | 2 | 1.555 | 0.156 |
| Error (*ε*) | 11988 | 18501 | 2 |  |  |

Note. The two-way ANOVA model is: *Υ_ijk_ = m + s_i_ +μ_j +_ (sμ)_ij +_ ε_ijk_*, where *Υ_ijk_*, *m*, *s_i_*, *μ_j_*, *(sμ)_ij_*, and ε_ijk_, respectively, represents coSNP*_O/E_*, the average of coSNP*_O/_*_E_, the selection effect with four-level factor (*s_1_* = -0.15; s_2_ = -0.1; *s_3_* = -0.05; *s_4_* = -0.01), the mutation effect with three-level factor (*μ_1_* = 1×10^-8^; *μ_2_* = 5×10^-8^; *μ_3_* = 1×10^-7^), the interaction effect between selection and mutation, and error with *k* =1,…, 1000 replicate.

**Supplemental Table S6.** Functional annotation clustering analysis of genes with coSNP*_i_*_=0_. The top two annotation clusters ranked by enrichment scores are listed.

| **Annotation** | **Term** | ***P*-value** | **Corrected *P*-value^*^** |
| --- | --- | --- | --- |
| Annotation cluster 1 (enrichment score: 11.58) | |  |  |
| PIR_SUPERFAMILY | RIRSF003152:G protein-coupled olfactory receptor, class II | 1.8E-20 | 1.7E-17 |
| SP_PIR_KEYWORDS | olfaction | 1.4E-19 | 3.7E-17 |
| INTERPRO | olfactory receptor | 2.0E-19 | 5.3E-16 |
| GOTERM_MF_FAT | Olfactory receptor activity | 1.5E-18 | 2.2E-15 |
| KEGG_PATHWAY | Olfactory transduction | 9.5E-18 | 1.8E-15 |
| GOTERM_BP_FAT | Sensory perception of smell | 1.6E-17 | 6.7E-14 |
| SP_PIR_KEYWORDS | Sensory transduction | 2.5E-16 | 3.5E-14 |
| GOTERM_BP_FAT | Sensory perception of chemical stimulus | 1.7E-15 | 3.4E-12 |
| PIR_SUPERFAMILY | PIRSF800006:rhodopsin-like G protein-coupled receptors | 4.7E-12 | 2.2E-9 |
| GOTERM_BP_FAT | Sensory perception | 1.0E-11 | 1.4E-8 |
| SP_PIR_KEYWORDS | g-protein coupled receptor | 1.4E-10 | 1.4E-8 |
| INTERPRO | GPCR, rhodopsin-like superfamily | 1.5E-9 | 2.0E-6 |
| INTERPRO | 7TM GPCR, rhodopsin-like | 2.0E-9 | 1.8E-6 |
| GOTERM_BP_FAT | cognition | 4.8E-9 | 4.9E-6 |
| SP_PIR_KEYWORDS | transducer | 7.5E-9 | 6.5E-7 |
| SP_PIR_KEYWORDS | receptor | 8.3E-9 | 6.5E-7 |
| GOTERM_BP_FAT | G-protein coupled receptor protein signaling pathway | 2.8E-6 | 1.7E-3 |
| GOTERM_BP_FAT | Neurological system process | 6.6E-6 | 3.4E-3 |
| SP_PIR_KEYWORDS | Cell membrane | 6.9E-6 | 3.8E-4 |
| Annotation cluster 2 (enrichment score: 8.9) | |  |  |
| UP_SEQ_FEATURE | glycosylation site: N-linked (GlcNAc) | 7.3E-22 | 2.7E-18 |
| SP_PIR_KEYWORDS | glycoprotein | 1.2E-21 | 4.8E-19 |
| UP_SEQ_FEATURE | topological domain: Extracellular | 6.2E-13 | 1.1E-9 |
| UP_SEQ_FEATURE | Topological domain: Cytoplasmic | 1.6E-11 | 2.4E-8 |
| GOTERM_CC_FAT | Plasma membrane | 6.2E-6 | 2.1E-3 |
| UP_SEQ_FEATURE | Transmembrane region | 1.5E-5 | 3.4E-3 |
| SP_PIR_KEYWORDS | transmembrane | 1.8E-5 | 9.3E-4 |
| GOTERM_CC_FAT | Integral to membrane | 1.4E-4 | 1.8E-2 |
| GOTERM_CC_FAT | Intrinsic to membrane | 6.9E-4 | 4.1E-2 |

^*^ The *P* values were adjusted using the Benjamini-Hochberg correction.

**Supplemental Figure S1.** The procedure of identifying chimpanzee SNPs in CE6.

**Supplemental Figure S2.** The coSNP*_O/E_* ratios of zero-fold, two-/three-fold, and four-fold degenerate nucleotides are determined by the comparisons between human SNPs derived from nine individuals[^1^](#_ENREF_1) and the chimpanzee SNPs analyzed in this study (excluding SNPs located within CpG dinucleotides). The full list of identified coSNPs is publicly available at http://treeslab1.genomics.sinica.edu.tw/coSNPs.html.

**(A)**

**Observed numbers of chimpanzee SNPs**

**(cont.)**

**Observed numbers of *Homo-Pan* coSNPs**

**(cont.)**

**(B)**

**Projected numbers of chimpanzee SNPs**

**(cont.)**

**Projected numbers of *Homo-Pan* coSNPs**

**(cont.)**

**(C)**

**Supplemental Figure S3.** Estimation of the coSNP*_O/E_* ratios based on the coSNPs determined by the comparisons between human SNPs (dbSNP138) and each of the five chimpanzee SNP datasets used (CE6, CE12, CW5, CW10, and CW25 SNPs), when the number of chimpanzee individuals exceeded 1,000. (A) Observed numbers, with fitted log-linear models and coefficients of determination (*R^2^*). (B) Projected numbers of chimpanzee SNPs and *Homo-Pan* coSNPs at the indicated sample sizes, using the fitted model in (A). (C) The coSNP*_O/E_* ratios for different types of i-fold degenerate sites and intronic sequences, according to the simulated chimpanzee SNPs and *Homo-Pan* coSNPs.

**(A)**

**(B)**

**Supplemental Figure S4.** Motif analysis based on the (A) Weblogo3 and (B) MEME predictions around the identified coSNPs at zero-fold (*i*=0), two-/three-fold (*i*=2 or 3), and four-fold (*i*=4) degenerate sites. (A) Motif logos showing the frequencies scaled according to the information content at each position (from -3 to +3) relative to the coSNPs with 95% confidence intervals. (B) The MEME motifs around the coSNPs (within -50 nucleotides to +50 nucleotides of the examined sites). Only the motifs supported by >100 sequences were listed. Bit values range from 0 to 2, with higher values indicating higher degrees of conservation.

**(A) (B)**

**Supplemental Figure S5.** Distribution of coSNP density of zero-fold (*i*=0), two-/three-fold (*i*=2 or 3), and four-fold (*i*=4) degenerate nucleotides in the 1M-bp windows (see the text) of different levels of (A) SNP density and (B) average recombination rate.


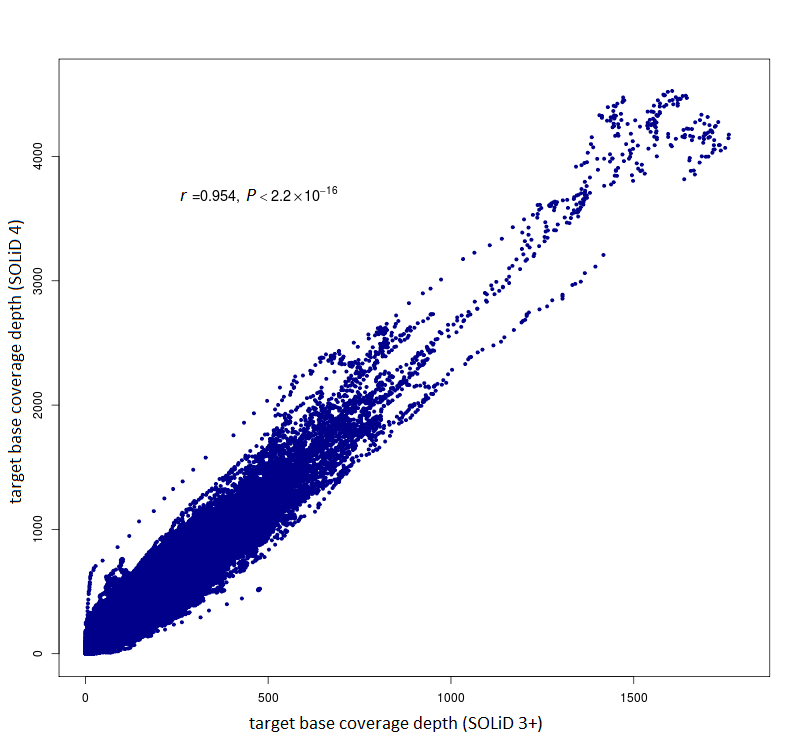


**Supplemental Figure S6.** Pearson's correlation between read depths of SOLiD 3+ and SOLiD 4 data derived from the same sample (chimpanzee individual ID: 20050256B10; see Supplemental Table S1).

(**A)**


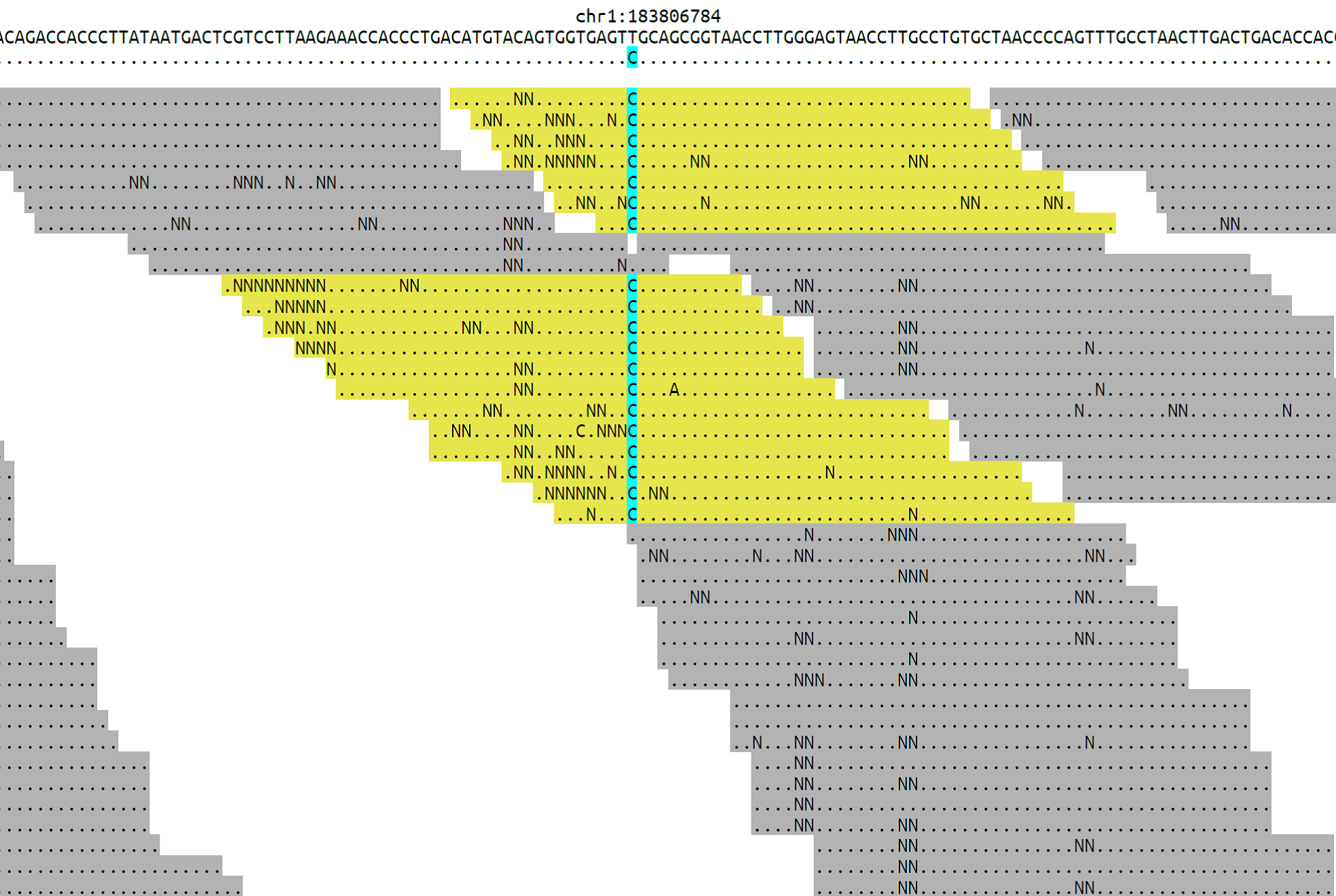
**(B)**

**Supplemental Figure S7.** Examples of false positive SNPs arising from equivocal read-to-genome alignments: (A) the called variant is not supported by any right-half parts of reads; and (B) none of the right-half parts of reads supporting the called variant are qualified, as each part contains both the called variant and other variant(s)/mismatch(es). The called variant is highlighted in blue. The reads supporting the variant are highlighted in yellow; the other reads around the called variant are highlighted in gray. For each read, dots and "N"s denote the nucleotides of exact matches and uncertain mismatches, respectively.

**Supplemental Figure S8.** Derived allele frequency distribution of the identified nonsynonymous SNPs in CE6.

**Supplemental References**

1 Prado-Martinez, J. *et al.* Great ape genetic diversity and population history. *Nature* **499**, 471-475 (2013).

2 Gokcumen, O. *et al.* Primate genome architecture influences structural variation mechanisms and functional consequences. *Proceedings of the National Academy of Sciences of the United States of America* **110**, 15764-15769 (2013).
